# Risk Assessment in Diffuse Large B-Cell Lymphoma by Combining Baseline Metabolic Tumor Volume and Peking Criteria When Evaluating Series ^18^F-Fluorodeoxyglucose Positron Emission Tomography Scans

**DOI:** 10.3389/fonc.2022.876581

**Published:** 2022-04-21

**Authors:** Tingting Yuan, Yuewei Zhang, Xuetao Chen, Maomao Wei, Hua Zhu, Yuqin Song, Zhi Yang, Jun Zhu, Xuejuan Wang

**Affiliations:** ^1^ Key Laboratory of Carcinogenesis and Translational Research (Ministry of Education/Beijing), NMPA Key Laboratory for Research and Evaluation of Radiopharmaceuticals (National Medical Products Administration), Department of Nuclear Medicine, Peking University Cancer Hospital and Institute, Beijing, China; ^2^ Key Laboratory of Carcinogenesis and Translational Research (Ministry of Education), Department of Lymphoma, Peking University Cancer Hospital and Institute, Beijing, China

**Keywords:** metabolic tumor volume, Peking criteria, positron emission tomography, diffuse large B-cell lymphoma, risk assessment

## Abstract

This study aimed to determine the predictive and prognostic value of baseline metabolic tumor volume (MTV) and the Peking criteria from serial positron emission tomography (PET) scans in diffuse large B-cell lymphoma, including 300 newly diagnosed patients who were prospectively treated with 2–4 cycles of standard first-line treatment (*clinicaltrials.gov identifier: NCT02928861*). PET/computed tomography (CT) examinations were performed at baseline, after two (PET-2) or four cycles (PET-4). PET during the interim was evaluated using Deauville 5-point scales (5-PS), ΔSUV_max_ criteria, and the Peking criteria which interpreted based on the maximum standard uptake of the liver (SUV_max-liver_). Peking criteria had better accuracy, positive predictive value (PPV), and specificity than other two methods. The MTV and Peking criteria both significantly predicted progression-free survival (PFS) and overall survival (OS). An MTV > 191 cm^2^ and Peking criteria of PET-2 and PET-4 > 1.6-fold SUV_max-liver_ was used as the cutoff for a positive result. PET-4 achieved higher accuracy, PPV, and specificity for 2-year PFS (83.3%, 86.7%, and 98.4%, respectively) and OS (92.6%, 73.3%, and 97.2%, respectively) than PET-2. Various prognostic models containing different risk factors were established *via* Cox regression analysis. The MTV and PET-2/PET-4 results were used to categorized patients into low-risk, intermediate-risk, and high-risk prognostic groups (with 0, 1, and 2 risk factors, respectively) (*P* < 0.0001). High burden MTV and positive PET-2 and PET-4 (>1.6-fold SUV_max-liver_) could identify high-risk patients with 2-year PFS and OS of 0.0% and 26.3% (95% confidence interval [CI]: N/A to 54.3%). When PET-2 and PET-4 were evaluated by 5-PS, the 2-year PFS and OS from high risk patients of three-parameters model achieved 31.4% (95%CI: 6.9%–55.9%) and 42.7% (95%CI: 14.6%–70.7%). In conclusion, combining baseline MTV and any regular response on PET/CT evaluated using the Peking criteria can improve prognostic value. Serial PET/CT from baseline MTV to PET-4 may have relatively greater predictive power for poor prognosis in diffuse large B-cell lymphoma.

**Clinical Trial Registration:**

ClinicalTrials.gov, identifier (NCT02928861).

## Introduction

Diffuse large B-cell lymphoma (DLBCL) is the largest subtype of non-Hodgkin lymphoma worldwide. Standard treatment with rituximab, cyclophosphamide, doxorubicin, vincristine, and prednisone (R-CHOP) can cure most cases of DLBCL, but approximately 30% will have treatment failure and a short survival period (1, 2). The most universally used prognostic index of DLBCL in clinical practice is the International Prognostic Index (IPI), which has 5 independent risk factors for survival: age (≤60 vs. >60 years), Ann Arbor stage (I/II vs. III/IV), number of extranodal (EN) sites (0–1 vs. ≥2), performance status (PS; 0–1 vs. ≥2), and serum lactate dehydrogenase (LDH; normal vs. elevated). However, this cannot dynamically evaluate disease status.

Molecular imaging with ^18^F-fluorodeoxyglucose positron emission tomography/computed tomography (^18^F-FDG PET/CT) is recommended for staging and assessing treatment efficacy. Metabolic tumor volume (MTV), interim PET, and end-of-treatment PET have been shown to predict outcomes in DLBCL (3–5). Patients with high MTV have a higher risk for disease progression or death than those with a low tumor burden. However, MTV only determines the patients’ risk at baseline, similar to the IPI. Two studies have suggested that PET-based prognostic models, including baseline MTV and PET scan after two cycles (PET-2), can help identify patients who can benefit from treatment adjustments or new treatments (6, 7). Both of those studies focused on the use of baseline and interim PET after two cycles of chemotherapy to predict therapeutic outcomes. However, studies have not yet explored the value of baseline PET combined with PET works after four cycles of therapy or serial PET scans.

Deauville 5-point scales (5-PS) and the change in maximum standardized uptake (ΔSUV_max_) method have been widely recognized in PET/CT image interpretation for the prognosis of DLBCL patients (8, 9). Previous studies by our research group suggested that the Peking criteria may be superior to those aforementioned methods (10–12), but those were retrospective studies with relatively small sample sizes. The present study aimed to identify the predictive and prognostic value of the Peking criteria and fully illustrate the clinical role of ^18^F-FDG PET/CT at baseline until four cycles of treatment.

## Methods

### Study Design

This was a single-center study. From January 2017 to December 2019, this study prospectively included 300 newly diagnosed DLBCL patients who underwent baseline PET/CT. Patients with a measurable lesion (i.e., minimum SUV_max_ of 2.5 in PET/CT) were included. All patients received 6–8 cycles of a first-line CHOP-like chemotherapy regimen: cyclophosphamide, 750 mg/m^2^ intravenous (i.v.) on day 1 (D1); vincristine, 1.4 mg/m^2^ i.v. on D1; doxorubicin, 50 mg/m^2^ i.v. on D1; and prednisolone, 60 mg/m^2^ orally on D1-5. This regimen was taken with or without rituximab (375 mg/m^2^ i.v. on D1). Treatment will not be changed on the basis of PET-4 scans unless there was definite progression. Patients with secondary malignant disease, serious infection, inflammation (e.g., HIV), or severe hepatic or renal dysfunction were excluded. The study was approved by the institutional review board at Peking University Cancer Hospital (2017KT23) in accordance with the Declaration of Helsinki *(clinicaltrials.gov identifier: NCT02928861)*.

### PET Image Acquisitions and Analysis

Patients were administered 3.7 MBq/kg ^18^F-FDG fast for at least 6 h before injection to ensure that the serum glucose level was less than 10 mmol/L. The PHILIPS EBW workstation was used for the imaging evaluation. Liver uptake was assessed along the largest cross-section. Interim PET scans were always performed before the third or fifth cycle. PET-2 was performed 17 ± 4 days after cycle 2 and PET-4 was about 17 ± 5 days after cycle 4. PET images were review by two blinded experienced nuclear physicians. SUV_max_ and MTV were measured in 3D mode using vendor-provided software. MTV was calculated by involving 41% SUV_max_ thresholding of automatically detected regions (SUV_max_ > 2.5). The total MTV of all lesions was also calculated in the baseline PET/CT scans. In the Peking criteria, an SUV_max_ greater than 1.6-fold that of the SUV_max_ of the liver (SUV_max-liver_) on PET-2 and PET/CT examination at PET-4 is used to identify patients with worse response and prognosis (10–12). In routine clinical practice, 5-PS score > 3 (^18^F-FDG uptake higher than in the liver) is considered positive when using the 5-PS to evaluate PET response (13). The percentage change in SUV_max_ between baseline and PET-2/PET-4 scans was described by ΔSUV_max_. In a previous study on DLBCL, the cutoff values of ΔSUV_max_ for PET-2 and PET-4 were reported to be 66% and 70%, respectively (4, 14).

### Survival Analysis and Statistical Analysis

Two-year progression-free survival (PFS) and overall survival (OS) were used as endpoints to evaluate the prognostic significance of interim PET/CT in the prospective cohort. PFS was defined as the time between the date of biopsy results and progression, relapse, or death from any cause. Overall survival (OS) was calculated from the date of biopsy results until death or last follow-up. Receiver operating characteristic (ROC) curve analysis was performed for continuous variables to determine optimal cutoff values for baseline MTV. Survival was analyzed using the Kaplan–Meier method and compared using the log-rank test. The life-table method was used to calculate 2-year PFS and OS rates. The multivariate Cox proportional hazards model was used for univariate and multivariate analyses to identify significant prognostic factors for survival outcomes. All statistical analyses were carried out using the SPSS software (version 25.0) and the survival package in R, version 4.1.2. Two-sided *P*-values less than 0.05 were considered statistically significant.

## Results

### Patient Characteristics

We initially enrolled 361 DLBCL patients from January 2017 and December 2019. Then, 61 patients were excluded due to secondary malignant disease (n = 6), no follow-up PET/CT (n = 34), no regular PET/CT examination (n = 12), no CHOP-like treatment (n = 1) and incomplete follow-up (n = 8). There were a total of 300 patients with complete data, who were thus eligible for analysis (Online **Supplementary Figure 1**). The median age was 57 years, with an Eastern Cooperative Oncology Group scale performance status of 0, 1, 2, 3, and 4 in 76.0% (228/300), 17.0% (51/300), 5.4% (16/300), 1.3% (4/300), and 0.3% (1/300) of patients, respectively. Meanwhile, Ann Arbor stage I, II, III, and IV disease was seen in 31 (10.3%), 109 (36.3%), 35 (11.7%), and 125 (41.7%) patients, respectively. IPI scores of 0–2 and 3–5 were documented in 206 (68.7%) and 94 (31.3%) patients, respectively. The baseline characteristics of DLBCL patients are shown in **Table 1**. 3.7%(11/300) of patients treated with first-line CHOP-like without rituximab due to elevated hepatitis B virus DNA content (n = 8), high level of hepatitis C virus RNA content (n = 1), and financial reasons (n = 2).

**Table 1:** Clinical features of patients with diffuse large B-cell lymphoma.

Parameters	No.	%
Total	300	
Age, years		
Median	58	
Range	18-83	
Sex		
Male	172	51.7
Female	127	42.3
Ann Arbor Stage		
I-II	140	46.7
III-IV	160	53.3
B symptoms		
Yes	99	33.0
No	201	67.0
Performance Status		
< 2	279	93.0
≥ 2	21	7.0
IPI score		
0–2	206	68.7
3–5	94	31.3
Lactate dehydrogenase		
Normal	154	51.3
Elevated	146	48.7
Bulky disease		
Yes	75	25.0
No	225	75.0
Baseline PET	300	
MTV > 191m^2^	110	36.7
MTV ≤ 191m^2^	190	63.3
Interim PET-2	252	
positive	35	13.8
negative	217	86.1
Interim PET-4	236	
positive	26	11.0
negative	210	89.0

IPI, International Prognostic Index; MTV, baseline metabolic tumor volume; PET-2, PET scan after two cycles of therapy; PET-4, PET scan after four cycles of therapy.

### PET/CT Features

All 300 eligible patients underwent initial pretreatment PET/CT. Among them, 252 patients underwent interim PET-2, while 236 were able to undergo PET-4. A total of 211 patients had a complete assessment with baseline scans, interim PET-2 scans, and interim PET-4 scans. A total of 43 patients received the following interventions: involved field radiotherapy for bulky disease (n = 24), high-dose methotrexate for CNS-directed prophylaxis strategies (n = 7), and autologous stem cell transplantation (n = 12).

Lymphoma lesions with an SUV_max_ higher than 1.6-fold SUV_max-liver_ on PET-2 and PET-4 were positive when using Peking criteria. A cutoff value of 191 cm^3^ was found to be optimal for MTV and was highly predictive of outcomes (PFS: *P* < 0.0001; OS: *P* < 0.0001). The areas under the ROC curves were 0.69 (*P* < 0.001) for PFS and 0.73 (*P* < 0.001) for OS. Three different image interpretation methods (i.e., Peking criteria, 5-PS, ΔSUV_max_ criteria) were used to assess the predictive and prognostic value of interim (2-year PFS and OS). Consistent with our group previous studies, the Peking criteria had superior accuracy, PPV, and specificity for both PFS and OS in PET-2 and PET-4 compared to the 5-PS and ΔSUV_max_ criteria (**Tables 2**, **3**). When baseline (i.e., MTV) and interim PET (i.e., PET-2, PET-4) were taken together, PET after treatment was classified as positive with an MTV > 191 cm^2^ and PET-2 or PET-4 > 1.6-fold SUV_max-liver_ (i.e., according to the Peking criteria). In this study, defined as consistently positive or negative, the combined strategy had better accuracy, PPV, NPV and specificity than the three image interpretation methods alone, as well as greater sensitivity than the Peking criteria, Deauville 5-point scales, and ΔSUV_max_ criteria. The combined method was evidently a stronger evaluation tool of DLBCL treatment. The accuracy, PPV and specificity (83.3%, 86.7%, and 98.4%, respectively for 2-year PFS; 92.6%, 73.3%, and 97.2%, respectively for 2-year OS) of MTV (> 191 cm^3^) combined with PET-4 (> 1.6-fold SUV_max-liver_) were higher than those of PET-2 (80.9%, 75.0%, and 95.3%, respectively for 2-year PFS; 89.0%, 50.0% and 92.2%, respectively for 2-year OS; **Table 4**).

**Table 2:** Accuracy of interim 18F-FDG PET/CT scan after two cycles of therapy (PET-2) interpreted using different image interpretation methods (n = 252).

Definition of iPET+	TN/FN/TP/FP(*n*)	Sensitivity	Specificity	PPV	NPV	Accuracy
**2-year PFS**						
Peking criteria (> 1.6-fold SUV_max-liver_)	163/54/24/11	30.8%	93.7%	68.6%	75.1%	74.2%
Deauville 5-point scales (> 3)	130/41/37/44	47.4%	74.7%	45.7%	76.0%	66.3%
ΔSUV_max_ criteria (< 66%)	151/53/25/23	32.1%	86.8%	52.1%	74.0%	69.8%
**2-year OS**						
Peking criteria (> 1.6-fold SUV_max-liver_)	196/21/14/21	40.0%	90.3%	40.0%	90.3%	83.3%
Deauville 5-point scales (> 3)	155/16/19/62	54.3%	71.4%	23.5%	90.6%	69.1%
ΔSUV_max_ criteria (< 66%)	183/21/14/34	40.0%	84.3%	29.2%	89.7%	78.2%

TN, true-negative; FN, false-negative; TP, true-positive; FP, false-positive. PPV, positive predictive value; NPV, negative predictive value; PFS, progression-free survival; OS, overall survival.

**Table 3:** Accuracy of interim ^18^F-FDG PET/CT scan after four cycles of therapy (PET-4) interpreted using different image interpretation methods (n = 236).

Definition of iPET+	TN/FN/TP/FP(*n*)	Sensitivity	Specificity	PPV	NPV	Accuracy
**2-year PFS**						
Peking criteria (> 1.6-fold SUV_max-liver_)	167/43/20/6	31.8%	96.5%	76.9%	79.5%	79.2%
Deauville 5-point scales (> 3)	148/37/26/25	41.3%	85.6%	51.0%	80.0%	73.7%
ΔSUV_max_ criteria (< 66%)	149/41/22/24	34.9%	86.1%	47.8%	78.4%	72.5%
**2-year OS**						
Peking criteria (> 1.6-fold SUV_max-liver_)	194/16/11/15	40.7%	92.8%	42.3%	92.4%	86.9%
Deauville 5-point scales (> 3)	173/12/15/36	55.6%	82.8%	29.4%	93.5%	79.7%
ΔSUV_max_ criteria (< 66%)	176/14/13/33	48.2%	84.2%	28.3%	92.6%	80.1%

TN, true-negative; FN, false-negative; TP, true-positive; FP, false-positive. PPV, positive predictive value; NPV, negative predictive value; PFS, progression-free survival; OS, overall survival.

**Table 4:** Accuracy of ^18^F-FDG PET/CT interpreted using baseline metabolic tumor volume combined with the Peking criteria.

Definition of iPET+	Patients (*n*)	TN/FN/TP/FP (*n*)	Sensitivity	Specificity	PPV	NPV	Accuracy
**2-year PFS**							
Combined MTV (>191 cm^2^) and PET-2 (>1.6-fold SUV_max-liver_) †	173/252 (68.7%)	122/27/18/6	40.0%	95.3%	75.0%	81.9%	80.9%
Combined MTV (>191 cm^2^) and PET-4 (>1.6-fold SUV_max-liver_) †	162/236 (68.6%)	122/25/13/2	34.2%	98.4%	86.7%	83.0%	83.3%
**2-year OS**							
Combined MTV (>191 cm^2^) and PET-2 (>1.6-fold SUV_max-liver_) †	173/252 (68.7%)	142/7/12/12	63.2%	92.2%	50.0%	95.3%	89.0%
Combined MTV (>191 cm^2^) and PET-4 (>1.6-fold SUV_max-liver_) †	162/236 (68.6%)	139/8/11/4	57.9%	97.2%	73.3%	94.6%	92.6%

TN, true-negative; FN, false-negative; TP, true-positive; FP, false-positive; PPV, positive predictive value; NPV, negative predictive value; PFS, progression-free survival; OS, overall survival. MTV, baseline metabolic tumor volume; SUV_max-liver_, maximum standard uptake of the liver; †PET interpretation is positive if both baseline MTV > 191 cm^2^ and Peking criteria > 1.6-fold SUV_max-liver_, PET interpretation is negative if negative with both methods.

### Treatment Outcome

**Figure 1:**
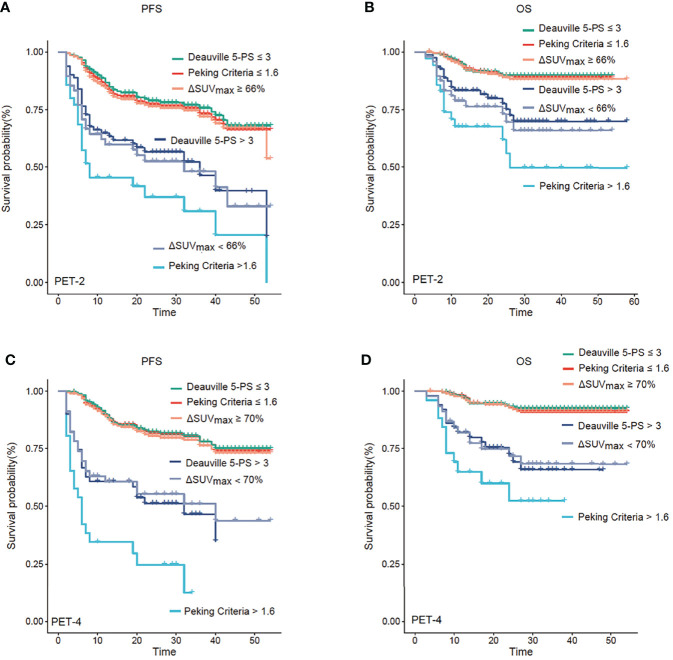
Progression-free survival (PFS) and overall survival (OS) analysis. Kaplan–Meier analysis of PFS and OS according to positron emission tomography (PET) after two cycles of therapy (PET-2) **(A, B)** and after four cycles of therapy (PET-4) **(C, D)**. PET-2 and PET-4 investigated by the three image interpretations were strong prognostic factors for both PFS and OS (all *P* < 0.001). The Peking criteria was superior 5-PS and ΔSUV_max_ criteria in terms of predicting prognosis.

The median follow-up was 28 months (range, 3-58 months). Kaplan–Meier curves showed that PET-2 and PET-4, as investigated by the 3 image interpretations, were strong prognostic factors for both PFS and OS (**Figure 1**, all *P*-values < 0.001). Since the sample size of patients treated with CHOP/CHOP-like without rituximab is rather small, no significant differences were found in survival outcomes when compared with patients treated with R-CHOP/R-CHOP-like (*P* = 0.749 for PFS, *P* = 0.203 for OS).

Univariate analysis of the primary cohort suggested that among the features of lymphoma patients, Ann Arbor stage, IPI, LDH, MTV, PET-2, and PET-4 were significant predictors of both PFS and OS (Online **Supplementary Table 1**). Prognostic model 1 was used to demonstrate that the MTV and PET-2 interpreted using the Peking criteria (since it was found to be superior to other image interpretations) were independent prognostic factors for both PFS (MTV: hazard ratio [HR] = 1.848, *P* = 0.027; PET-2: HR = 4.001, *P* < 0.001) and OS (MTV: HR = 3.517, *P* = 0.005; PET-2: HR = 5.025, *P* < 0.001) *via* multivariate analysis (**Table 5**). This PET/CT-related prognostic index for the DLBCL model was able to efficiently identify 3 categories of patients with different outcomes in the PET-2 group (n = 252; Online **Supplementary Figure 2**). A total of 149 (59.1%) patients were classified as low-risk (no risk factor), 79 (31.4%) as intermediate-risk (1 risk factor; HR = 2.748 for PFS, HR = 4.803 for OS), and 24 (9.5%) as high-risk (2 risk factors; HR = 27.500, HR = 17.213 for OS) in the interim PET-2 group when using Peking criteria. The 2-year PFS and OS, respectively, according to category were as follows: low-risk: 83.9% (95% CI: 77.5% to 90.4%) and 94.4% (95% CI: 90.3% to 98.4%); intermediate-risk: 52.8% (95% CI: 40.8% to 64.8%) and 74.6% (95% CI: 63.4% to 85.8%); and high-risk: 27.5% (95% CI: 7.0% to 48.0%) and 37.7% (95% CI: 12.4% to 62.9%). As 5-PS was commonly used in clinical practice for PET efficacy assessments of lymphoma (8), risk assessment by combining MTV and PET-2 according to 5-PS was still workable (Online **Supplementary Figure 2**). The 2-year PFS and OS of low-risk, intermediate-risk, and high-risk were 84.9% vs. 55.6% vs. 45.2% and 95.4% vs. 75.2% vs. 59.3% (*P* < 0.0001; Online **Supplementary Table 2**).

**Table 5:** Multivariate analysis for survival and disease progression (PET response interpretation: Peking criteria).

Risk factor	Progression-free survival			Overall survival			
	HR	95% CI	P	HR		95% CI	P
**Model 1** (MTV + PET-2, n = 252)							
MTV (positive vs. negative)	1.848	1.072–3.186	0.027		3.517	1.462–8.459	0.005
PET-2 (positive vs. negative)	4.001	2.208–7.251	<0.001		5.025	2.162–11.681	<0.001
**Model 2** (MTV + PET-4, n = 236)							
MTV (positive vs. negative)	2.027	1.201–3.420	0.008		3.670	1.542–8.732	0.003
PET-4 (positive vs. negative)	7.964	4.602–15.153	<0.001		6.753	2.930–15.566	<0.001
**Model 3** (MTV+ PET-2 + PET-4, n = 211)							
MTV (positive vs. negative)	1.817	1.055–3.130	0.031		3.420	1.436–8.140	0.005
PET-2 (positive vs. negative)	1.853	0.905–3.793	0.091		2.700	1.025–7.112	0.044
PET-4 (positive vs. negative)	6.145	3.017–12.515	<0.001		4.327	1.653–11.324	0.003
**Model 4** (n = 211)							
PET-2 (positive vs. negative)	——	——	0.134		2.733	1.003–7.445	0.049
PET-4 (positive vs. negative)	10.054	5.535–18.262	<0.001		4.430	1.637–11.989	0.003
LDH (normal vs. abnormal)	3.240	1.794–5.850	<0.001		5.339	1.808–15.768	0.002

HR, hazard ratio; CI, confidence interval; MTV, baseline metabolic tumor volume, MTV > 191 cm^2^ is considered positive; SUV_max-liver_: maximum standard uptake of the liver; PET-2 positive: Peking criteria method of PET scan after two cycles (PET-2) of therapy >1.6-fold SUV_max-liver_; PET-4 positive: Peking criteria method of PET scan after four cycles (PET-4) of therapy > 1.6-fold SUV_max-liver_.

Prognostic model 2 tested MTV and PET-4; both of which were found to be independent prognostic factors of PFS and OS (**Table 5**). Patients with low baseline MTV and good response had favorable prognosis regardless of treatment (2-year PFS of 84.0%; 2-year OS of 93.3%). Meanwhile, patients with either high baseline MTV or positive PET response (according to the Peking criteria) had an intermediate prognosis (2-year PFS of 64.1%; 2-year OS of 79.8%). The interim PET-4 group included 147 (62.3%), 74 (31.3%), and 15 (6.4%) low-risk, intermediate-risk, and high-risk patients, respectively. According to the log-rank test, this model showed that patients with a high baseline tumor burden (MTV > 191 cm^3^) and poor response (PET-4 > 1.6-fold SUV_max-liver_) had significantly poorer prognosis (2-year PFS of 5.8%; 2-year OS of 42.9%; *P* < 0.0001; Online **Supplementary Table 3**). Patients with 5-PS > 3 point and high baseline MTV had 2-PFS of 31.3% and 2-year OS of 47.8%, lower than low-risk, intermediate-risk patients (*P* < 0.0001; Online **Supplementary Figure 3**).

**Figure 2:**
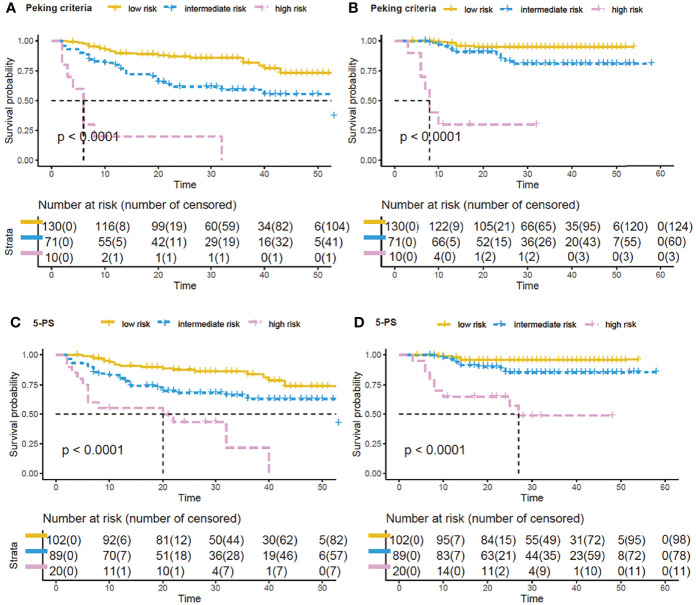
Survival curves by new model of three PET parameters by using Peking criteria or Deauville 5-point scales (5-PS). Kaplan–Meier analysis of progression-free survival (PFS) and overall survival (OS) by the new prognostic model by combining baseline metabolic tumor volume (MTV), PET after two cycles of therapy (PET-2) and PET after four cycles of therapy (PET-4): low-risk, no risk factor; intermediate-risk, 1 or 2 risk factors; high-risk, 3 risk factors. With Peking criteria, the respective 2-year PFS and OS of the 3 groups were 85.3% (95% CI, 79.2%–91.4%) and 94.6% (95% CI, 90.3%–98.8%) for low-risk patients (yellow curve); 58.6% (95% CI, 46.1%–71.0%) and 79.4% (95% CI, 68.2%–90.6%) for intermediate-risk patients (blue curve); and 0.0% and 26.3% (95% CI, N/A to 54.3%) for high-risk patients (purple curve) **(A, B)**. For 5-PS, 2-year probabilities of low-, intermediate-, and high-risk groups were 85.7%, 65.1%, 31.4% for PFS **(C)**, and were 95.5%, 84.1%, 42.7% for OS **(D)**.

Prognostic model 3 involved MTV-2, PET-2 and PET-4, which were available for 211 patients. Only PET-2 was not an independent factor for 2-year PFS. Similar to the previous models, patients were again classified into the same three categories of risk (**Figure 2**). The three-parameter risk-stratification model was able to statistically predict both PFS and OS (both *P* < 0.001). The 2-year PFS and OS rates of high-risk cases were 0.0% and 26.3% (95% CI: N/A to 54.3%), respectively. When using 5-PS to help to determine high-risk patients, there were 31.4% for 2-year PFS and 42.7% for 2-year OS (*P* < 0.0001; **Figure 2**).

Model 4 in **Table 5** included all individual factors that were significant on univariate analysis. Only PET-4 and LDH were identified as independent prognostic indicators for PFS and OS (both *P* < 0.001), whereas PET-2 did not reach statistical significance for PFS (*P* = 0.134).

## Discussion

This study explored the use of serial PET/CT from baseline to the interim treatment response as a tool for risk assessment. The Peking criteria outperformed the 5-PS and ΔSUV_max_ methods in predicting the prognosis of 2-year PFS and OS. Approximately 30.3% of DLBCL patients experienced disease progression, and 14.3% died at the time of last follow-up.

**Figure 3:**
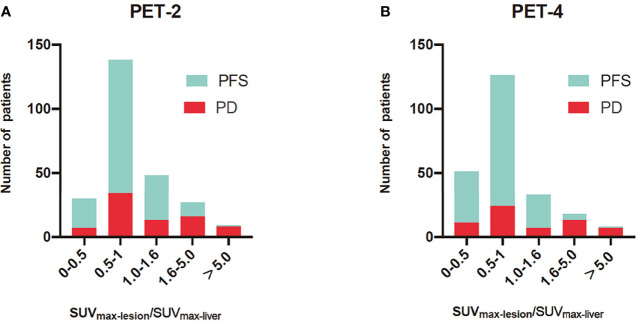
Histograms of SUV_max-lesion_/SUV_max-liver_ for progression-free survival (PFS) and progression disease (PD) in PET after two cycles of therapy (PET-2) **(A)** and PET after four cycles of therapy (PET-4) **(B)**. The SUV_max-lesion_/SUV_max-liver_ was SUV_max_ ratio between the tumor and liver. For PET-2 and PET-4, interim PET was evaluated by 1.6-fold of SUV_max_-liver in the Peking criteria and 1.0-fold of SUV_max-liver_ in Deauville 5-point scales. The Peking criteria was better than 5-PS in distinguishing false positive lesions.

Mamot et al. reported that interim PET/CT scans could not predict 2-year OS when PET/CT assessments were categorized by using 5-PS (4 and 5 vs. 1–3) in DLBCL patients treated with R-CHOP-14 (15). The positivity rates of PET-2 and PET-4 (60.1% and 43.2%) were significantly higher than those in prior studies when using ΔSUV_max_, ranging from 11% to 21.9% (4, 16, 17). In accordance with the evaluation of ΔSUV_max_, PET-2 and PET-4 had positivity rates of 13.8% and 11.0%, respectively, when assessed using the Peking criteria. The high positivity rates of R-CHOP-14 therapy may be due to the short time interval (less than 14 days) between chemotherapy administration and the PET/CT scan, with nonspecific FDG uptake, which is caused by treatment-related inflammation and bone remodeling. The interval between last chemotherapy and PET examination was about 16 to 21 days in other studies. The Peking criteria (1.6-fold SUV_max-liver_ for PET-2 and PET-4) and ΔSUV_max_ both outperformed 5-PS (1.0-fold SUV_max-liver_) in distinguishing false positive lesions (11, 18). There was considerable proportion of PFS patients whose lymphoma residual site SUV_max_ was between 1.0- to 1.6-fold that of the SUV_max-liver_; these cases were deemed to have poor prognosis upon image interpretation *via* the 5-PS (**Figure 3**). Kurch et al. also suggested that to screen patients for more aggressive therapies, a cutoff between scores of 4 and 5 on the visual scale (5-PS) may be more beneficial. The qPET method, which is based on a single scan (similar to the 5-PS and the Peking criteria), was found to include essentially the same scan information as ΔSUV_max_ (9). However, the qPET method is slightly more complicated to calculate than the Peking criteria and 5-PS.

In this trial, MTV and PET-2/PET-4 had independent predictive value. A prognostic model was proposed based on these features derived from PET/CT imaging that depict two different aspects of the disease: tumor burden and treatment response. High risk patients identified by Peking criteria had more unfavorable prognosis than groups evaluated by using 5-PS. An interim PET/CT at a later time point (PET-4) might be able to better distinguish between patients with good or poor prognosis than PET-2 (**Table 4**). Prognostic model 2 combined the Peking criteria (PET-4 > 1.6-fold SUV_max-liver_) and MTV (> 191 cm^2^), and was able to identify high-risk patients with poor prognosis. Regarding survival, 8 out of 15 high-risk patients died within 8 months. Meanwhile, most survivors (6 out of 7) in the high-risk category from the prospective PET-4 groups changed their therapeutic regimen after 4 or 6 cycles of first-line CHOP-like chemotherapy, with the longest overall regimen lasting 38 months. Only one patient received autologous stem cell transplantation. Combining baseline MTV and PET-2/PET-4 evaluated by the Peking criteria, we identified 9 (90.0%) high-risk patients who had disease progression within 32 months, whereas 7 (70%) patients died in less than 11 months.

Previous studies showed that combining MTV and PET-2 can identify patients with poor prognosis and guide clinical treatment (6, 7). Schmitz et al. defined three groups by combining MTV and PET-2 response (cutoff of ΔSUV_max_: 66%), with low-risk, intermediate-risk, and high-risk groups having respective 2-year probabilities of 90.9%, 62.5%, 29.9% for PFS and 95.5%, 77.4%, and 37.1% for OS (6), which were similarly to our three-parameter risk-stratification model by combined with the Peking criteria. Our results revealed that the combination of MTV and PET-4 was still effective. In line with this, Yuan et al. found that performing an interim ^18^F-FDG PET/CT after one cycle of chemotherapy is feasible and yields similar predictive results compared to PET-2 in DLBCL patients (3). Numerous studies have shown that interim PET could predict the prognosis of a different magnitude in patients suffering from DLBCL.

Patients receive PET/CT scans in various stages of cancer therapy for very different reasons. Physicians can take full advantage of interim regular PET/CT scans. We found that the combination of MTV and interim PET was optimal for evaluating prognosis. In the combined assessment, interim PET was considered positive if both the MTV and Peking criteria were positive. Our results suggest that patients with a large tumor burden that are positive on interim PET can be candidates for novel therapeutic approaches, such as immune checkpoint inhibitors and chimeric antigen receptor T cells. Further multicenter studies with a larger sample size are needed to determine the optimal combination of prognostic parameters for use in clinical practice.

In conclusion, serial ^18^F-FDG PET/CT scans from baseline to interim treatment are practical in evaluating the curative effect and prognosis of DLBCL. When taken together, assessing the MTV and interim response on PET/CT using the Peking criteria enhances the prognostic value of PET, and appears promising to guide therapeutic strategies.

## Data Availability Statement

The original contributions presented in the study are included in the article/**Supplementary Material**. Further inquiries can be directed to the corresponding author.

## Ethics Statement

The studies involving human participants were reviewed and approved by Peking University Cancer Hospital. The patients/participants provided their written informed consent to participate in this study.

## Author Contributions

Concept and design: TY and XW. Literature search: TY, YZ, and XC. Data acquisition, data analysis: TY, YZ, and XW. Manuscript preparation: TY, YZ, and XW. Manuscript editing: TY, XC, MW, and XW. Manuscript review: YZ, MW, HZ, YS, ZY, JZ, and XW. All authors contributed to the article and approved the submitted version.

## Funding

This work was supported by the National Natural Science Foundation of China (grant number.82071957), Beijing Hospitals Authority Clinical Medicine Development of special funding support (grant number XMLX202120) and Capital’s Funds for Health Improvement and Research (grant number 2018-2-1024).

## Conflict of Interest

The authors declare that the research was conducted in the absence of any commercial or financial relationships that could be construed as a potential conflict of interest.

## Publisher’s Note

All claims expressed in this article are solely those of the authors and do not necessarily represent those of their affiliated organizations, or those of the publisher, the editors and the reviewers. Any product that may be evaluated in this article, or claim that may be made by its manufacturer, is not guaranteed or endorsed by the publisher.
