# Supplementary material for: Risk Assessment in Diffuse Large B-Cell Lymphoma by Combining Baseline Metabolic Tumor Volume and Peking Criteria When Evaluating Series 18F-Fluorodeoxyglucose Positron Emission Tomography Scans
Source: Front Oncol. 2022 Apr 21;12:876581. doi: 10.3389/fonc.2022.876581 (PMC9069109; doi:10.3389/fonc.2022.876581)
Supplement: Supplementary file 1 [file DataSheet_1.docx]

Supplementary Material

**Supplementary Table 1. Univariate analysis for survival and disease progression (n = 211)**

|  | **Progression-free survival** | | |  | **Overall survival** | | | |
| --- | --- | --- | --- | --- | --- | --- | --- | --- |
| **Risk factor** | **HR** | **95% CI** | ***P*** |  | **HR** | **95% CI** | ***P*** |  |
| Sex (male vs. female) | 0.844 | 0.503–1.416 | 0.520 |  | 0.550 | 0.235–1.285 | 0.168 |  |
| Age (>60ys vs. ≤60ys) | 2.164 | 1.286–3.641 | 0.004 |  | 2.840 | 1.215–6.643 | 0.016 |  |
| Ann Arbor stage (Ⅰ/Ⅱ vs. Ⅲ/Ⅳ) | 3.160 | 1.756–5.684 | <0.001 |  | 5.036 | 1.721–14.741 | 0.003 |  |
| B symptoms (yes vs. no) | 1.338 | 0.793–2.259 | 0.275 |  | 2.138 | 0.960–4.760 | 0.063 |  |
| Performance status (<2 vs. ≥2) | 1.482 | 0.635–3.459 | 0.363 |  | 2.700 | 0.921–7.911 | 0.070 |  |
| IPI (0-2 vs. 3-5) | 2.726 | 1.633–4.551 | <0.001 |  | 4.315 | 1.887–9.869 | 0.001 |  |
| LDH (normal vs. abnormal) | 2.530 | 1.482–4.318 | 0.001 |  | 6.638 | 2.267–19.435 | 0.001 |  |
| Bulky disease (yes vs. no) | 2.108 | 1.242–3.579 | 0.006 |  | 3.054 | 1.366–6.825 | 0.007 |  |
| MTV (positive vs. negative) | 2.499 | 1.497–4.172 | <0.001 |  | 4.760 | 2.037–11.125 | <0.001 |  |
| PET-2 (positive vs. negative) | 5.041 | 2.877–8.832 | <0.001 |  | 7.095 | 3.134–16.060 | <0.001 |  |
| PET-4 (positive vs. negative) | 9.596 | 5.354–17.200 | <0.001 |  | 8.861 | 3.913–20.067 | <0.001 |  |

HR: hazard ratio; CI: confidence interval; MTV: baseline metabolic tumor volume, MTV > 191 cm^2^ is considered positive; SUV_max-liver_: maximum standard uptake of the liver; PET-2 positive: Peking criteria method of PET scan after two cycles (PET-2) of therapy > 1.6-fold SUV_max-liver_; PET-4 positive: Peking criteria method of PET scan after four cycles (PET-4) of therapy > 1.6-fold SUV_max-liver_.

**Supplementary Table 2. Survival and disease progression of three risk categories (PET response interpretation: Deauville 5-point scales)**

| **Risk factor** | **Patients (*n*)** | **Progression-free survival at 2-year** | | | **Overall survival at 2-year** | |
| --- | --- | --- | --- | --- | --- | --- |
|  |  | Cumulative Proportion (%) | 95% CI | | Cumulative Proportion (%) | 95% CI |
| **Model 1** (MTV + PET-2, n = 252) | |  |  | |  |  |
| low-risk | 129(51.2%) | 84.9 | 80.4–89.4 | | 95.4 | 91.5–99.3 |
| intermediate-risk | 73 (29.0%) | 55.6 | 43.3–67.9 | | 75.2 | 64.0–86.4 |
| high-risk | 50 (19.8%) | 45.2 | 29.1–61.3 | | 59.3 | 40.9–77.7 |
| **Model 2** (MTV + PET-4, n = 236) | |  |  | |  |  |
| low-risk | 131(55.5%) | 84.4 | 77.7–91.1 | | 94.3 | 89.8–98.8 |
| intermediate-risk | 81 (34.3%) | 66.3 | 55.5–77.1 | | 82.7 | 73.1–92.3 |
| high-risk | 24 (10.2%) | 31.3 | 10.1–52.5 | | 47.8 | 22.7–72.9 |
| **Model 3** (MTV + PET-2 + PET-4, n = 211) | | | |  |  |  |
| low-risk | 102 (48.3%) | 85.7 | 78.4–93.0 | | 95.5 | 91.2–99.8 |
| intermediate-risk | 89 (42.2%) | 65.1 | 54.1–76.1 | | 84.1 | 75.3–92.9 |
| high-risk | 20 (9.5%) | 31.4 | 6.9–55.9 | | 42.7 | 14.6–70.7 |

CI: confidence interval; Model 1 and model 2 combined two parameters: low-risk, no risk factor; intermediate-risk, 1 risk factor; high-risk, 2 risk factors; Model 3 combined three parameters: low-risk, no risk factor; intermediate-risk, 1 or 2 risk factors; high-risk, 3 risk factors; PET-2, PET scan after two cycles of therapy; PET-4, PET scan after four cycles of therapy.

**Supplementary Table 3. Survival and disease progression of three risk categories (PET response interpretation: Peking criteria)**

| **Risk factor** | **Patients (*n*)** | **Progression-free survival at 2-year** | | | | **Overall survival at 2-year** | | |
| --- | --- | --- | --- | --- | --- | --- | --- | --- |
|  |  | Cumulative Proportion (%) | 95% CI | | Cumulative Proportion (%) | | 95% CI | |
| **Model 1** (MTV + PET-2, n = 252) | |  |  | |  | |  | |
| low-risk | 149(59.1%) | 83.9 | 77.5–90.4 | | 94.4 | | 90.3–98.4 | |
| intermediate-risk | 79 (31.4%) | 52.8 | 40.8–64.8 | | 74.6 | | 63.4–85.8 | |
| high-risk | 24 (9.5%) | 27.5 | 7.0–48.0 | | 37.7 | | 12.4–62.9 | |
| **Model 2** (MTV + PET-4, n = 236) | |  |  | |  | |  | |
| low-risk | 147(62.3%) | 84.0 | 77.6–90.4 | | 93.3 | | 88.7–97.9 | |
| intermediate-risk | 74 (31.3%) | 64.1 | 53.1–75.1 | | 79.8 | | 68.8–90.8 | |
| high-risk | 15 (6.4%) | 5.8 | NA–20.3 | | 42.9 | | 16.9–68.8 | |
| **Model 3** (MTV + PET-2 + PET-4, n = 211) | | | |  | |  | |  |
| low-risk | 130 (61.6%) | 85.3 | 79.2–91.4 | | 94.6 | | 90.3–98.8 | |
| intermediate-risk | 71 (33.6%) | 58.6 | 46.1–71.0 | | 79.4 | | 68.2–90.6 | |
| high-risk | 10 (4.7%) | 0 | NA | | 26.3 | | NA–54.3 | |

CI: confidence interval; Model 1 and model 2 combined two parameters: low-risk, no risk factor; intermediate-risk, 1 risk factor; high-risk, 2 risk factors; Model 3 combined three parameters: low-risk, no risk factor; intermediate-risk, 1 or 2 risk factors; high-risk, 3 risk factors; PET-2, PET scan after two cycles of therapy; PET-4, PET scan after four cycles of therapy.


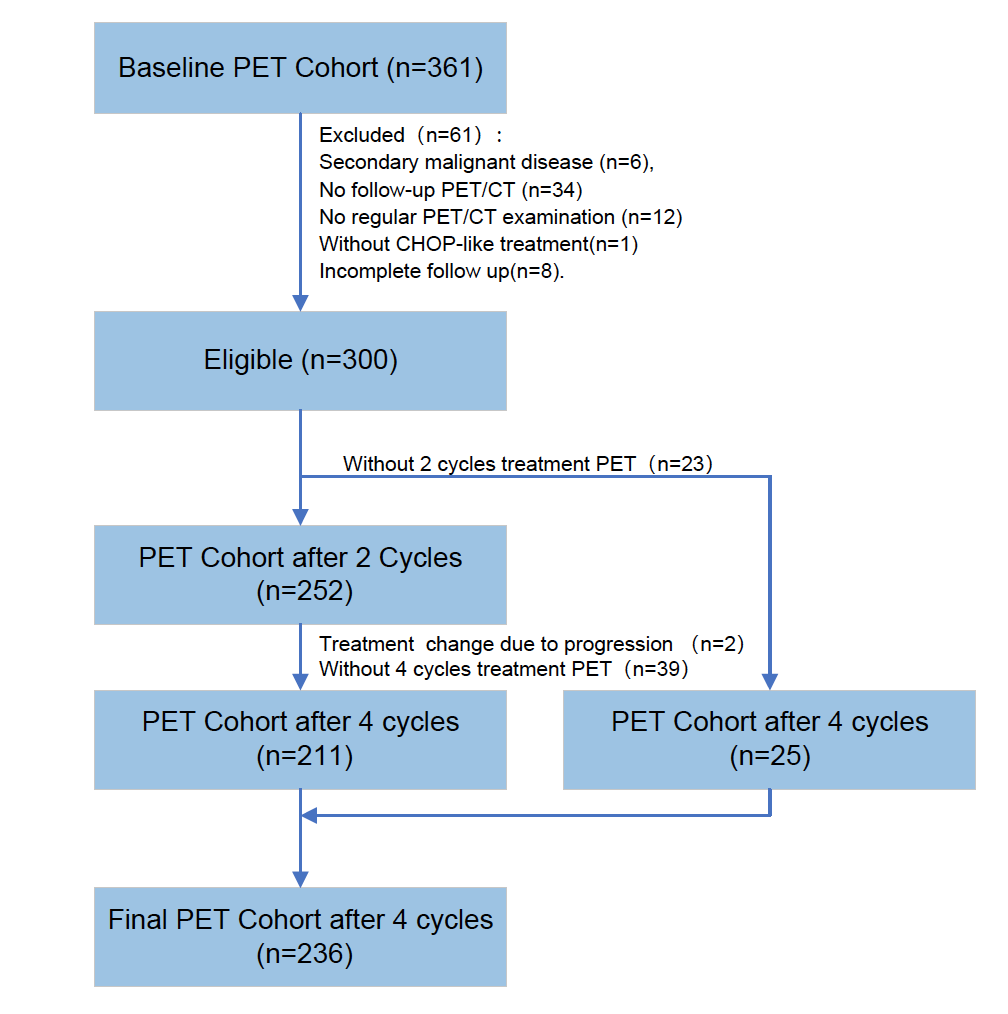


**Supplementary Figure 1.** **CONSORT diagram.** Sixty-one patients were excluded from the analyses, resulting in 300 evaluable patients.

**
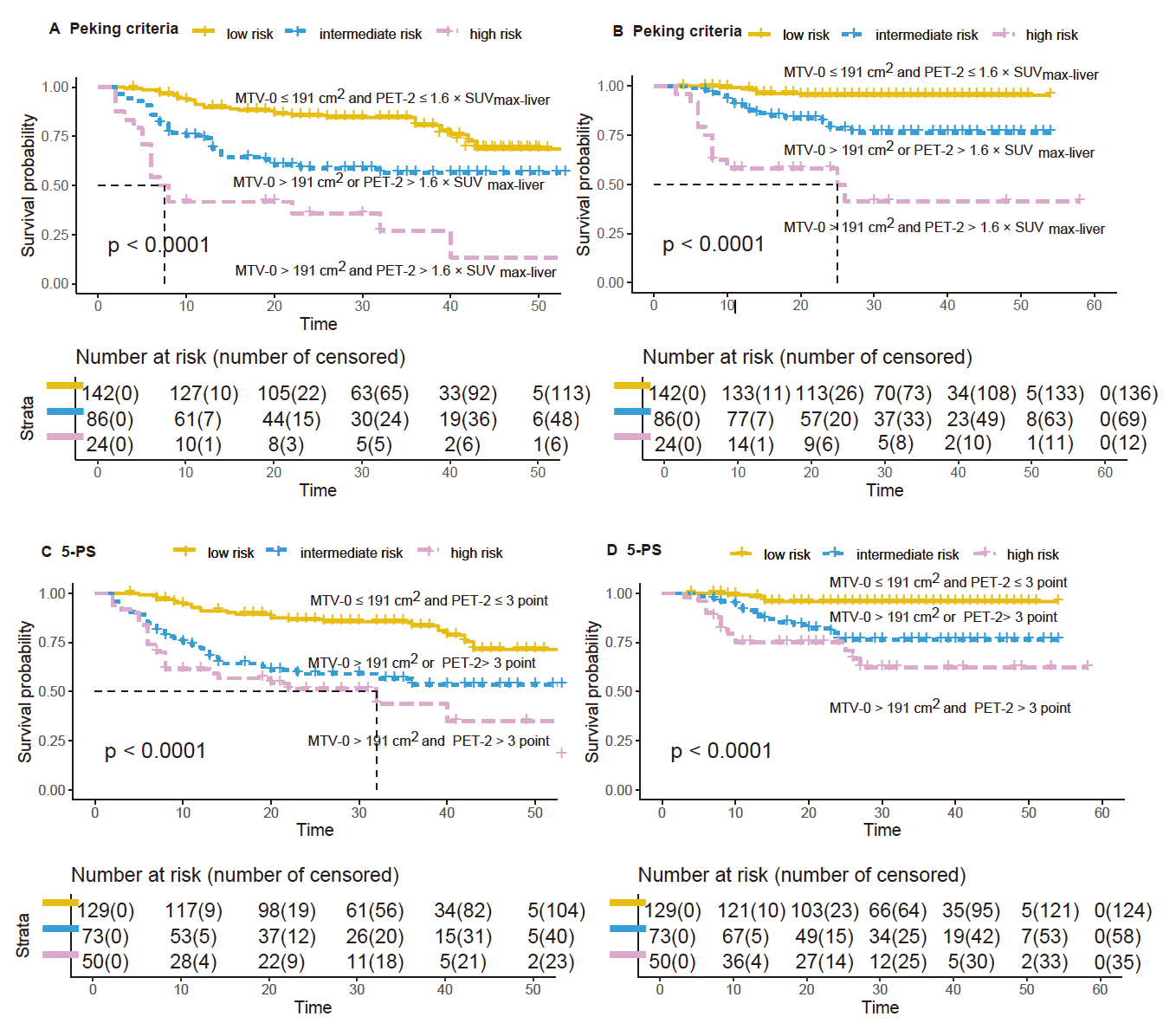
**

**Supplementary Figure 2. Kaplan–Meier analysis of progression-free survival (PFS) and overall survival (OS) according to baseline metabolic tumor volume (MTV) combined PET after two cycles of therapy (PET-2) by using Peking criteria (A, B) or Deauville 5-point scales (5-PS) (C, D).**

**
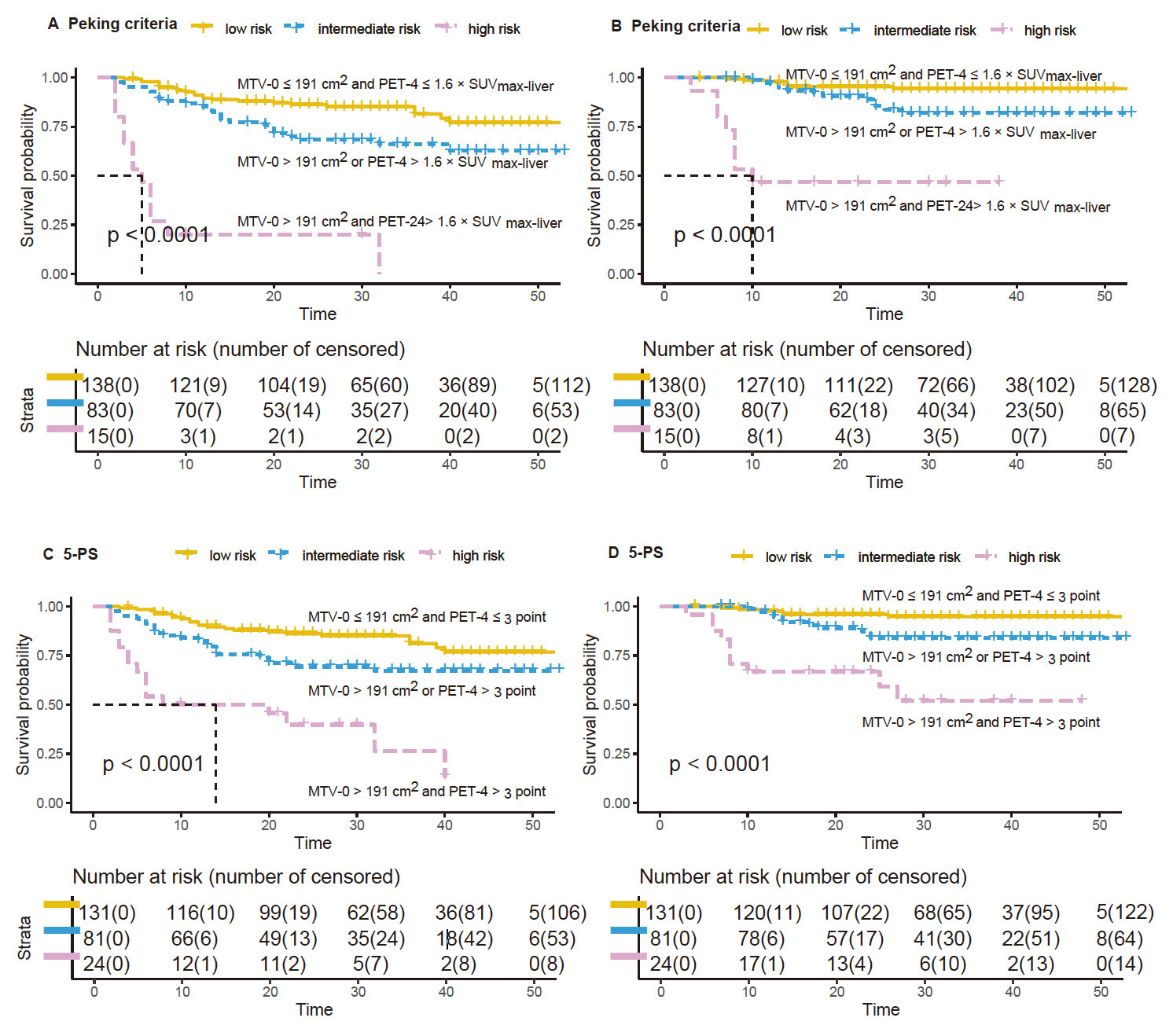
**

**Supplementary Figure 3. Kaplan–Meier analysis of progression-free survival (PFS) and overall survival (OS) according to baseline metabolic tumor volume (MTV) PET after four cycles of therapy (PET-4) by using Peking criteria (A, B) and Deauville 5-point scales (5-PS) (C, D).**
